# Apologetic

**Published:** 1878-10-20

**Authors:** 


### APOLOGETIC.

A portion of our September issue was improperly put together in the binding—a fact not discovered until quite a number had been mailed, and it is feared a few of the faulty ones among them. If so, those who recieved them can easily remedy the matter by clipping the stitches, and properly arranging the pages; or if they will send them back to us we will make the correction.

We regret these mistakes, but they will occur occasionally in all kinds of business; and we trust our friends will make due allowance. If they knew the difficulties encountered in Southern Publishing Houses we are sure they would not only excuse but sympathize with us.

Notwithstanding the obsticles to Journalism in the South we have the satisfaction to state that our Journal is improving; extending its circulation and becoming more and more the favorite of the busy practitioner.

Some delay in the issue of the present number has been caused by the unusual press of work in our Publishing House, by reason of the Fair and Rex carnival.

